# Comparison of expression patterns of six canonical clock genes of follicular phase and luteal phase in Small-tailed Han sheep

**DOI:** 10.5194/aab-64-457-2021

**Published:** 2021-10-28

**Authors:** Qi Han, Xiaoyun He, Ran Di, Mingxing Chu

**Affiliations:** Key Laboratory of Animal Genetics and Breeding and Reproduction of the Ministry of Agriculture and Rural Affairs, Institute of Animal Science, Chinese Academy of Agricultural Sciences, Beijing 100193, P.R. China

## Abstract

The circadian rhythm is a biological rhythm that is closely related to the rhythmic expression of a series of clock genes. Results from several studies have indicated that clock genes are associated with the estrous cycle in female animals. Until now, the relationship between estrus cycle transition and clock gene expression in reproductive-axis-related tissues has remained unknown in Small-tailed Han (STH) sheep. This study was conducted to analyze the expression patterns of six canonical clock genes (*Clock*, *BMAL1*, *Per1*, *Per2*, *Cry1*, and *Cry2*) in the follicle phase and luteal phase of STH sheep. We found that all six genes were expressed in the brain, cerebellum, hypothalamus, pituitary, ovary, uterus, and oviduct in follicle and luteal phases. The results indicated that *Clock* expression was significantly higher in the cerebellum, hypothalamus, and uterus of the luteal phase than that of the follicle phase, whereas *BMAL1* expression was significantly higher in the hypothalamus of the luteal phase than that of the follicle phase. *Per1* expression was significantly higher in the brain, cerebellum, hypothalamus, and pituitary of the luteal phase than that of the follicle phase, and *Per2* expression was significantly higher in the hypothalamus, pituitary, and uterus of the luteal phase than that of the follicle phase. *Cry1* expression was significantly higher in the brain, cerebellum, and hypothalamus of the luteal phase than that of the follicle phase, whereas *Cry2* expression was significantly higher in the pituitary of the luteal phase than that of the follicle phase. The clock gene expression in all tissues was different between follicle and luteal phases, but all clock gene mRNA levels were found to exhibit higher expression among seven tissues in the luteal phase. Our results suggest that estrous cycles may be associated with clock gene expression in the STH sheep. This is the first study to systematically analyze the expression patterns of clock genes of different estrous cycle in ewes, which could form a basis for further studies to develop the relationship between clock genes and the estrous cycle.

## 1 Introduction

Circadian rhythms are the nearly 24 h processes that allow an organism to coordinate appropriate physiological responses to the environmental light–dark changes associated with the rotation of the Earth (Goldstein and Smith, 2016). In mammals, various behaviors and physiological functions of the body can present a typical circadian rhythm, such as the sleep–wake cycle, food intake, body temperature fluctuation, hormone secretion, and energy metabolism (Chacon et al., 2004; Buhr et al., 2010; Mohawk et al., 2012). Like many other functional activities, animal reproduction is closely related to the circadian rhythm. Previous studies have shown that many aspects of the reproductive biology of males and females are regulated by the circadian rhythm (Brown-Grant et al., 1977; Peterlin et al., 2019; Mills and Kuohung, 2019), including the estrus cycle, levels of luteinizing hormone (LH), ovulation, production and maturation of sperm, fertilization, insemination, and embryo implantation (Gray et al., 1978; Christian et al., 2005). However, disruptions to the circadian rhythm have noticeable negative effects on female reproductive health, such as irregular ovulatory cycles, reduced fertility, increased miscarriage rates, and anomalous fetal development (Gotlieb et al., 2020). Moreover, studies in rodents have shown that ablation of the master circadian clock in the brain can block relevant clock output signals or disrupt the genes driving the circadian clock function at the cellular level, leading to pronounced deficits in ovulation and reproductive success (Gotlieb et al., 2020).

The circadian rhythm is controlled by the central clock in the hypothalamic suprachiasmatic nucleus (SCN) and the peripheral clocks in various tissues (Zhang et al., 2016). It is accepted wisdom that circadian rhythms are generated and maintained by an autoregulatory transcription–translation feedback loop consisting of clock genes and their protein products (Takahashi, 2015; Honma et al., 2018). Currently, more than 10 genes have been identified that form the basis of cellular rhythmicity in mammals, including two transcriptional activators, *Clock* and *BMAL1*, the transcriptional repressors called Period (*Per1*, *Per2*), and Cryptochrome (*Cry1*, *Cry2*) (Preitner et al., 2002; Leloup and Goldbeter, 2003), which are considered to be the core clock genes (Sen and Hoffmann, 2020). These genes contribute to reproductive processes in mammals (Pan et al., 2020). Multiple studies have indicated a connection between clock gene expression and reproduction. For example, clock gene expression is related to ovarian follicular development (Sen and Sellix, 2016; Nagao et al., 2019) and steroidogenesis (Liu et al., 2014; Sellix, 2015), and they can regulate LH surge to affect ovulation (Simonneaux et al., 2017). Studies on the mouse estrous ovary have shown that the expression of clock protein is rhythmic in four follicular stages (Wiggins and Legge, 2016). In addition, clock gene expression abnormalities resulted in increased rates of placental abruption later in pregnancy (Qiu et al., 2016). Studies in humans have suggested that disruption of the cellular clocks also perturbs reproductive cycles including ovulation (Mahoney, 2010).

Increasing reproductive efficiency with regard to litter size (also known as fecundity) is one of the economic objectives of the sheep industry. Most sheep breeds produce one lamb per gestation, and only a few produce twins, which substantially affects the overall reproductive efficiency. Compared with other sheep, the Small-tailed Han (STH) sheep is an excellent local breed in China, which is well known for high fecundity, especially year-round estrus, and an average lambing rate of 250 %. Accordingly, they are considered to be a good breeding source (Di et al., 2012; Wang et al., 2015). It is generally known that seasonal reproduction is regulated by the hypothalamus–pituitary–gonadal (HPG) axis system. However, the clock genes play important roles in regulating the HPG axis, especially the secretion of GnRH and LH (Chappell et al., 2003), hinting that the clock genes may have an effect on the change in the estrus pattern in animals. However, there are few reports about the functions of clock genes in sheep reproduction.

In the present study, to ascertain the potential role of *Clock*, *BMAL1*, *Per1*, *Per2*, *Cry1*, and *Cry2* in STH sheep, we compare and analyze the mRNA expression levels of these genes in HPG-related tissues between the follicular phase and luteal phase of STH. Our study paves the way for an in-depth study of the estrus mode transition of STH sheep.

## 2 Materials and methods

### 2.1 Selection of experimental sheep and sample collection

The six Small-tailed Han adult ewes (3 years old) used for study were selected from the Sheep & Goat Breeding Farm of Tianjin Institute of Animal Sciences (Tianjin, China). All sheep were kept in a sheltered outdoor paddock and were provided with alfalfa hay and concentrate, with clean water available ad libitum. All sheep were subjected to estrus synchronization administration of progesterone (CIDR device, InterAg Co., Ltd., New Zealand) for 12 d. Then three estrus-synchronized sheep were euthanized (intravenous pentobarbital at 100 mg per kilogram) within 45–48 h of CIDR removal (follicular phase); the remaining three estrus-synchronized sheep were euthanized (intravenous pentobarbital at 100 mg per kilogram) 9 d after CIDR removal (luteal phase). All animals were euthanized (intravenous pentobarbital at 100 mg per kilogram), and seven tissues (brain, cerebellum, hypothalamus, pituitary, ovary, uterus, oviduct) were collected from each animal. All tissues were snap-frozen in liquid nitrogen and then stored at -80 ${}^{\circ }$C to be used for RNA extraction.

All animals used in the present study were approved by the Science Research Department (in charge of animal welfare issues) of the Institute of Animal Science, Chinese Academy of Agricultural Sciences (IAS-CAAS; Beijing, P.R. China). Ethical approval was given by the animal ethics committee of IAS-CAAS (no. IAS2020-82, 28 July 2020).

### 2.2 Total RNA extraction and cDNA synthesis

Total RNA in the different tissues (each tissue smashed, mixed, and 50–100 mg used for RNA extraction) was extracted using the Trizol reagent according to the manufacturer's instructions (Invitrogen Inc., Carlsbad, CA, USA). The concentration and integrity of the RNA samples were detected by ultraviolet spectrophotometry (UV-1201, Shimadzu, Kyoto, Japan) and 1.5 % agarose gel electrophoresis (U = 160 V; 10 min). Then, total RNA (500 ng) was reverse-transcribed into cDNA using a PrimeScript™ RT reagent kit (TaKaRa Bio Inc., Dalian, China) following the method provided by the manufacturer. Briefly, each 10 µL reaction mix contained 5× PrimeScript Buffer (for real time) 2.0 µL, PrimeScript RT Enzyme 0.5 µL, oligo dT Primer 0.5 µL, random 6-mers 0.5 µL, and total RNA 500 ng, with ddH${}_{2}$O accounting for the rest of the volume. The PCR thermocycler program was as follows: 37 ${}^{\circ }$C for 15 min, followed by 85 ${}^{\circ }$C for 5 s. After reaction, the cDNA was stored at -20 ${}^{\circ }$C until use.

### 2.3 Primer design

The corresponding quantitative real-time polymerase chain reaction (qRT-PCR) primers were designed using Primer 5.0 (Palo Alto, CA, USA) software based on the GenBank sequence of target genes (*Clock, BMAL1, Per1, Per2, Cry1, Cry2*, and *RPL19*). All primers were synthesized by Beijing Tianyi Biotechnology Co., Ltd. (Beijing, China). The corresponding qRT-PCR primers are shown in Table 1.

### 2.4 QRT-PCR analysis for mRNA expression

QRT-PCR was performed to examine the expression levels of *Clock*, *BMAL1*, *Per1*, *Per2*, *Cry1*, and *Cry2* in seven tissues from the follicular phase and luteal phase of STH sheep. We performed qRT-PCR on a LightCycler480 system (Roche, Basel, Sweden). Each 10 µL qRT-PCR reaction mix contained 5 µL of TB Green Premix Ex Taq II (Tli RNaseH Plus) (TaKaRa Bio Inc., Dalian, China), 2 µL of cDNA, 0.4 µL of forward primer, and 0.4 µL of reverse primer, with ddH${}_{2}$O accounting for the rest of the volume. The PCR program consisted of initial denaturation at 95 ${}^{\circ }$C for 5 min, followed by 40 cycles of amplification at 95 ${}^{\circ }$C for 10 s, annealing at 60 ${}^{\circ }$C for 30 s, extension at 72 ${}^{\circ }$C for 60 s, a melting curve step (65–95 ${}^{\circ }$C, starting fluorescence acquisition at 65 ${}^{\circ }$C with measurements every 10 s to 95 ${}^{\circ }$C), and a final cooling step to 4 ${}^{\circ }$C. Three replicates were performed for each reaction, and mRNA levels were normalized to the expression level of the housekeeping gene *RPL19* in each sample.

**Table 1:** Primers used for real-time reverse transcription polymerase chain reaction.

Gene names	Primer sequence (5${}^{\prime }$–3${}^{\prime }$)	Length (bp)	$T_{m}$ (${}^{\circ }$C)	Accession no.
*Clock*	F: 5${}^{\prime }$-CAACGCACACATAGGCCTTC-3${}^{\prime }$ R: 5${}^{\prime }$-CTATTATGGGTGGTGCCCTGT-3${}^{\prime }$	181	60	NM_001130932.1
*BMAL1*	F: 5${}^{\prime }$-ATTGCAACCGGAAACGCAAG-3${}^{\prime }$ R: 5${}^{\prime }$-TGGTGGCACCTCGTAATGTT-3${}^{\prime }$	288	62	NM_001129734.1
*Per1*	F: 5${}^{\prime }$-GCCAGACAACCCTTCTACCAGT-3${}^{\prime }$ R: 5${}^{\prime }$- GGCTTGCACCTGCTTGACACA-3${}^{\prime }$	187	61	XM_027974931.1
*Per2*	F: 5${}^{\prime }$-TTACGACCACACATTCGCCA-3${}^{\prime }$ R: 5${}^{\prime }$-CCCCAGACTGCACGATCTTC-3${}^{\prime }$	171	61	XM_027967088.1
*Cry1*	F: 5${}^{\prime }$-ACAGGTGGCGATTTTTGCTT-3${}^{\prime }$ R: 5${}^{\prime }$-TCCAGCTTCAGTTGCCAGTT-3${}^{\prime }$	215	61	NM_001129735.1
*Cry2*	F: 5${}^{\prime }$-AGGCTGTTCAAGGAATGGGG-3${}^{\prime }$ R: 5${}^{\prime }$-CGTAGGTCTCATCGTGGCTC-3	316	61	NM_001129736.1
*RPL19*	F: 5${}^{\prime }$-ATCGCCAATGCCAACTC-3${}^{\prime }$ R: 5${}^{\prime }$-CCTTTCGCTTACCTATACC-3${}^{\prime }$	154	60	XM_012186026.1

### 2.5 Statistical analysis

All the experiments were repeated at least three times, and the results are shown as mean ± standard error of the mean (SEM). The relative gene expression levels were calculated based on the 2${}^{-\Delta \Delta \mathrm{Ct}}$ method (Livak and Schmittgen, 2001; Schmittgen and Livak, 2008). Statistical analysis was performed using SPSS 22.0 software (IBM Armonk, NY, USA) and GraphPad Prism 7.0 software (GraphPad Prism Software Inc., San Diego, CA, USA). The Student's t test was used to compare the levels of gene expression between two groups. To compare the means of more than two groups, one-way analysis of variance (ANOVA) was used followed by the Tukey' honest significant difference (HSD) test. Differences were considered statistically significant at P<0.05.

## 3 Results

### 3.1 Expression levels of *Clock*

**Figure 1:**
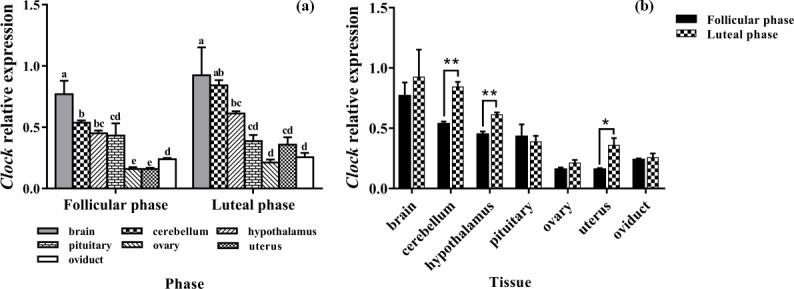
Comparison of expression of *Clock* among two phases **(a)** and among tissues **(b)**. Different letters mean significant difference (P < 0.05). One asterisk denotes differences at P<0.05, and two asterisks denote differences at P<0.01. Data are presented as the mean ± SEM.

As shown in Fig. 1a, *Clock* was expressed in seven tissues of the follicular and luteal phases in STH sheep, with the highest level being in the brain. In the follicular phase, the expression level of *Clock* in the brain was significantly higher than that in the cerebellum, hypothalamus, pituitary, ovary, uterus, and oviduct (P<0.05), and the expression level in the cerebellum was significantly higher than that in the pituitary, ovary, uterus, and oviduct (P<0.05). Furthermore, the expression levels of *Clock* in the ovary and uterus were lower than other tissues (P<0.05). In the luteal phase, there was no significant difference in the pituitary, ovary, uterus, and oviduct (P>0.05), but the expression levels of *Clock* in the ovary and oviduct were significantly lower than that in the brain, cerebellum, and hypothalamus (P<0.05), and *Clock* expression in the pituitary and uterus was significantly lower than that in the brain and cerebellum (P<0.05). In addition, the expression level of *Clock* in the cerebellum was not different from that in the brain and hypothalamus (P>0.05), but there was a significant difference between the brain and hypothalamus (P<0.05).

As depicted in Fig. 1b, there was no significant difference in the expression level of *Clock* in the brain, pituitary, ovary, and oviduct between two phases, but its expression in the cerebellum, hypothalamus, and uterus of the luteal phase was higher than that of the follicular phase (P<0.01, P<0.01, P<0.05).

### 3.2 Expression levels of *BMAL1*

**Figure 2:**
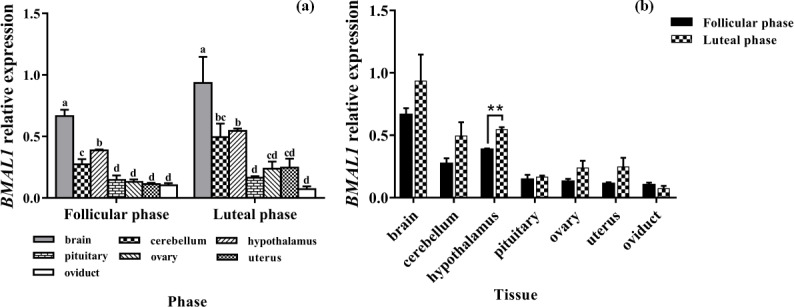
Comparison of expression of *BMAL1* among two phases **(a)** and among tissues **(b)**. Different letters mean significant difference (P<0.05). One asterisk denotes differences at P<0.05, and two asterisks denote differences at P<0.01. Data are presented as the mean ± SEM.

The results of the *BMAL1* analysis are shown in Fig. 2. *BMAL1* was expressed in seven tissues of the follicular and luteal phases in STH sheep, with the highest level being in the brain, followed by the hypothalamus, with a significant difference between the two tissues (P<0.05). And the expression levels of *BMAL1* in them were higher than that in the pituitary, ovary, uterus, and oviduct (P<0.05). Additionally, there was no significant difference among the pituitary, ovary, uterus, and oviduct (P>0.05) (Fig. 2a).

In addition, the tissue expression profiles of the two phases in STH sheep were analyzed, and the expression level of *BMAL1* in the hypothalamus of the luteal phase was significantly higher than that of the follicular phase (P<0.01); however, there was no significant difference in other tissues (P>0.05) (Fig. 2b).

### 3.3 Expression levels of *Per1*

**Figure 3:**
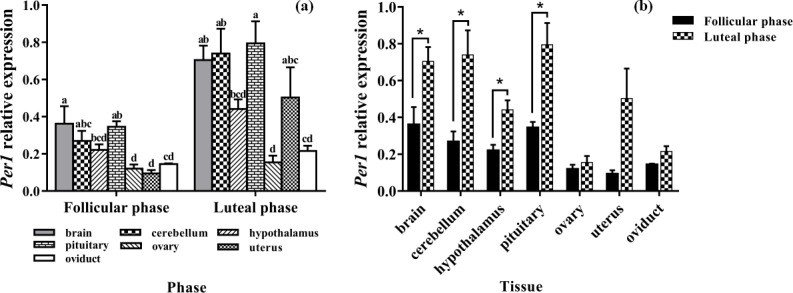
Comparison of expression of *Per1* among two phases **(a)** and among tissues **(b)**. Different letters mean significant difference (P < 0.05). One asterisk denotes differences at P<0.05, and two asterisks denote differences at P<0.01. Data are presented as the mean ± SEM.

As shown in Fig. 3a, *Per1* was widely expressed in all selected tissues. There was no difference in the expression of *Per1* in the brain, cerebellum, and pituitary, but the expression of *Per1* in them was significantly higher than that in the ovary and oviduct (P<0.05).

In the comparison between the follicular phase and luteal phase (Fig. 3b), the expression levels of *Per1* in the brain, cerebellum, hypothalamus, and pituitary were much higher in the luteal phase than in the follicular phase (P<0.05), but there was no significant difference in other tissues (P>0.05).

### 3.4 Expression levels of *Per2*

**Figure 4:**
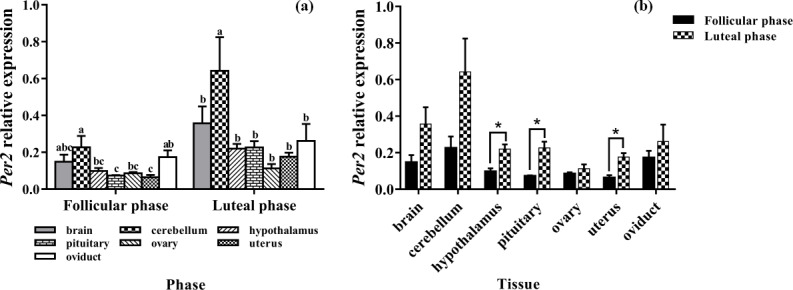
Comparison of expression of *Per2* among two phases **(a)** and among tissues **(b)**. Different letters mean significant difference (P<0.05). One asterisk denotes differences at P<0.05, and two asterisks denote differences at P<0.01. Data are presented as the mean ± SEM.

Subsequently, we evaluated the expression levels of *Per2* in seven tissues of the follicle and luteal phases. As depicted in Fig. 4a, *Per2* was expressed in all selected tissues, with the highest level being in the cerebellum. In the follicular phase, the expression levels of *Per2* in the brain, cerebellum, and oviduct had no significant difference (P>0.05), but its expression in the cerebellum was significantly higher than that in the hypothalamus, pituitary, ovary, and uterus (P<0.05). Furthermore, in the luteal phase, the expression level of *Per2* in the cerebellum was significantly higher than those of other tissues (P<0.05).

As shown in Fig. 4b, compared with the follicular phase, *Per2* expression levels of the luteal phase were significantly higher in the hypothalamus, pituitary, and uterus (P<0.05), but there was no difference in other tissues (P>0.05).

### 3.5 Expression levels of *Cry1*

**Figure 5:**
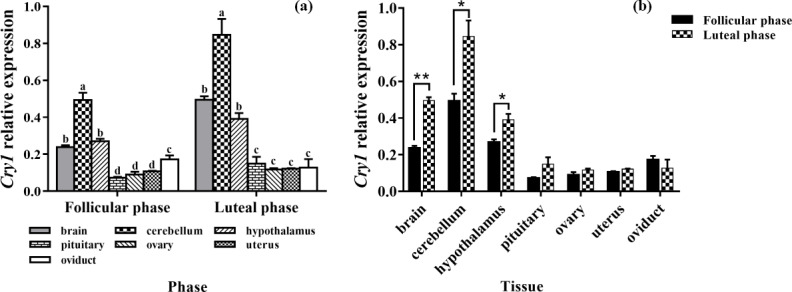
Comparison of expression of *Cry1* among two phases **(a)** and among tissues **(b)**. Different letters mean significant difference (P<0.05). One asterisk denotes differences at P<0.05, and two asterisks denote differences at P<0.01. Data are presented as the mean ± SEM.

As depicted in Fig. 5a, the mRNA expression of *Cry1* was detected in all tissues, with the highest level being in the cerebellum, and its expression was significantly higher than those of other tissues (P<0.05). In addition, the expression levels of *Cry1* in the brain and hypothalamus were significantly higher than that in the pituitary, ovary, uterus, and oviduct (P<0.05). As depicted in Fig. 5b, the expression levels of *Cry1* were markedly higher in the brain, cerebellum, and hypothalamus of the luteal phase than that of the follicular phase (P<0.01, P<0.05, P<0.05), but there was no difference in other tissues (P>0.05).

### 3.6 Expression levels of *Cry2*

**Figure 6:**
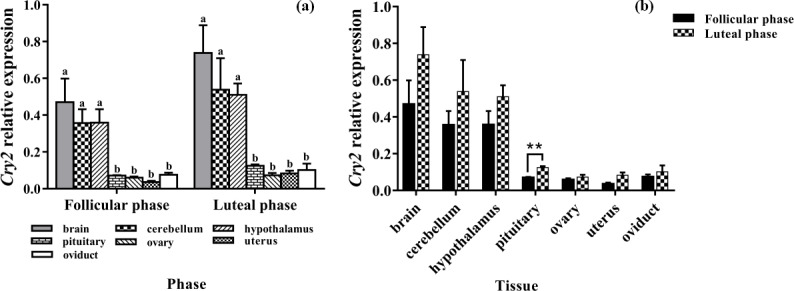
Comparison of expression of *Cry2* among two phases **(a)** and among tissues **(b)**. Different letters mean significant difference (P < 0.05). One asterisk denotes differences at P<0.05, and two asterisks denote differences at P<0.01. Data are presented as the mean ± SEM.

Figure 6a clearly shows that the expression pattern of *Cry2 *in the seven tissues was similar in the follicular phase and luteal phase. *Cry2* is expressed among the seven tissues, with the highest level being in the brain. In addition, the expression levels of *Cry2* in the brain, cerebellum, and hypothalamus were significantly higher than that in the pituitary, ovary, uterus, and oviduct (P<0.05). As shown in Fig. 6b, the *Cry2* expression level of the luteal phase was significantly higher in the pituitary than that of the follicular phase (P<0.01), but there was no difference in other tissues (P>0.05).

## 4 Discussion

The *Clock* was the first mammalian circadian clock gene to be discovered (King et al., 1997). Accumulating evidence indicates that *Clock* plays an important role in female reproduction. For example, *Clock* mutant mice showed irregular estrous cycles, with no normal LH surge on the day of proestrus, as well as failing to show circadian rhythms of clock gene expression in the uterus (Dolatshad et al., 2006). In addition, prior studies have indicated that *Clock*–*Clock* mutant mice have differences in pregnancies, with a higher rate of fetal absorption, serious dystocia, morphological abnormalities, and lower serum progesterone and estradiol levels (Miller and Takahashi, 2014; Pilorz et al., 2018). Thus, *Clock* is an important gene in female reproduction. In the present study, we found that the *Clock* gene was expressed in all the selected tissues. This result is consistent with that of previous studies, which shows that *Clock* mRNA expression is not limited to the SCN but lies in diverse tissues (King et al., 1997). However, the highest expression of *Clock* in the brain of STH sheep differs from reports on mice; this discrepancy may be attributed to the differences between species and sampling time points. Furthermore, the expression of *Clock* in the uterus in the luteal phase was found to be significantly higher than in the follicular phase, which may be beneficial to pregnancy.

Previous research has also demonstrated that *BMAL1* is necessary to maintain normal female reproduction (Brown et al., 2012; Wang et al., 2017). *BMAL1* null mice showed irregular estrous cycles, late onset of puberty, absence of proestrus LH surges, implantation failure, and progesterone-dependent implantation failure (Boden et al., 2010, 2013). Additionally, studies on mice and porcine luteinized granulosa cells in vitro have suggested that *BMAL1* knockdown reduces transcriptional levels of steroidogenesis-associated genes and deteriorates P4 and E2 production, with promoting granulosa cell apoptosis (Wang et al., 2017, 2020). Therefore, the *BMAL1* gene has a certain impact on follicle development and female reproduction. In the current study, our results demonstrate that *BMAL1* is expressed among seven tissues, implicating *BMAL1* as involved in STH sheep reproduction. Recently, as physiologically verified by immunohistochemistry, *BMAL1* expression significantly increased during mouse corpus luteum formation (Wiggins et al., 2016). Likewise, Kobayashi et al. (2018) found that *BMAL1* expression reached a maximum 16 h after hCG (human chorionic gonadotropin) administration when follicle luteinization occurred. These results suggest a role of *BMAL1* in ovarian luteinization. Consistent with these previous reports, our present data demonstrate that the expression levels of *BMAL1* were higher in the brain, cerebellum, hypothalamus, pituitary, ovary, and uterus of the luteal phase compared with the follicular phase. However, there is only a significant difference in the hypothalamus. One potential explanation is that the differences in the genetic models or sampling position led to these results.

*Period* genes are a class of circadian clock genes that act as transcriptional repressors, forming a core component of the circadian clock (Dibner and Schibler, 2018). Evidence from previous studies has suggested that mice deficient in *Per1* or *Per2* had a short circadian period. Additionally, double mutations of *Per1*–*Per2* genes in mice disrupted circadian rhythms in locomotor activity and the expression of key clock genes as well as clock-regulated genes (Zheng et al., 1999, 2001). However, *Per3* mutation mice did not show any effect on the circadian rhythm, implying that *Per3* is considered to be unnecessary for maintaining the circadian rhythm (Shiromani et al., 2004). Therefore, *Per1* and *Per2* were selected for the investigation of the STH sheep in this study. Previous studies reported that *Per1* and *Per2* are widely expressed throughout the body (Nakamura et al., 2005; Lamont et al., 2007). Further studies revealed that the expression of *Per1* and *Per2* was detected in many tissues of Sunite sheep (Xiang et al., 2019a, b). Our data also show that *Per1* and *Per2* are expressed in the brain, cerebellum, and hypothalamic–pituitary–gonadal axis. And we found that *Per1* and *Per2* are more highly expressed in the hypothalamus and pituitary of the luteal phase than that of the follicle phase, whereas the hypothalamus and pituitary play central roles in the production and release of reproductive hormones. Additionally, a previous study reported that *Per1* and *Per2* mRNA may participate in the coordination of GnRH (gonadotropin-releasing hormone) and LH (luteinizing hormone) surge (Zheng et al., 2019). This research implies that *Per1* and *Per2* may have a certain effect on STH sheep reproduction. Of course, further research is needed in this regard.

In mammals, the *Cryptochrome* gene family has two members, *Cry1* and *Cry2*, which are negative feedback regulators of the circadian clock (Duong et al., 2011). *Cry1* knockout mice present a short-period circadian rhythm at behavioral and tissue as well as cell levels, whereas *Cry2* knockout mice exhibit a completely opposite phenotype (van der Horst et al., 1999). The reason behind these opposing phenotypes is still unclear. In addition, double mutations of *Cry1* and *Cry2* genes in mice resulted in complete loss of the circadian rhythm (van der Horst et al., 1999). This research indicated that *Cry1* and *Cry2* are key for producing and maintaining the circadian rhythm for the body. Previous studies have shown that *Cry1* is widely expressed in the human and mouse heart, ovary, and testis (Kobayashi et al., 1998). Subsequently, studies on sheep demonstrated that the *Cry1* gene was expressed in pituitary tissues at different ages (Zhan et al., 2012). Next, Gao et al. found that *Cry1* was expressed in the hypothalamus–pituitary–ovary axis, suggesting that it may initiate estrus and seasonal reproduction (Gao et al., 2013). Likewise, the expression of *Cry1* mRNA and protein was also detected in the male yak reproductive axis (Chen et al., 2019). Consistent with previous studies, our results show that *Cry1* is expressed in all selected tissues of STH sheep, implying that *Cry1* is closely related to sheep reproduction. To date, the *Cry2* gene has only been shown to be involved in the reproduction of diapausing animals through the seasons (Pan et al., 2020). In this study, we also found that *Cry2* was expressed in all selected tissues of STH sheep. The results suggest that *Cry2* may play a role in sheep reproduction. However, what part *Cry2* plays in reproduction remains largely unclear, and more work will have to be done to identify this exact role in the future.

Circadian rhythms in physiology and behavior are known to be influenced by the estrous cycle in female rodents (Nakamura et al., 2010), so we investigated the expression of six clock genes in different tissues of follicular and luteal phases in STH sheep. The present study suggests that six clock genes are expressed in all selected tissues of STH sheep with different expression levels, which may play an important role in maintaining various physiological and behavioral rhythms of sheep, especially the reproductive function, but their specific role needs to be further studied. And all six clock gene mRNA levels were found to exhibit higher expression among seven tissues in the luteal phase, which is consistent with the findings in female rats by Nakamura et al. (2010). Likewise, a study performed using the female cynomolgus model demonstrated that significant differences were found in the expression of *Cry1* or *Per2* mRNA between the late follicular phase and mid-luteal phase (Xu et al., 2015). Additionally, studies on rodents and monkeys have suggested that progesterone levels are associated with changes in clock gene expression during the estrus cycle. Further studies found that P4, but not E2, acutely induces clock gene expression in MCF-7 human cancer cells (Nakamura et al., 2010). It is well known that the progesterone level in the luteal phase is much higher than that in the follicular phase in sheep, implying that the different expression levels of clock genes in the follicular phase and luteal phase of STH sheep is probably caused by progesterone levels. Moreover, clock gene knockout mice have all been shown to have deficiencies in embryonic implantation and pregnancy maintenance (Pilorz and Steinlechner, 2008). These data indicate that high expression of clock genes during the luteal phase may be the natural demand of embryonic implantation and early development during the luteal phase. Previous studies have suggested that the total amount of plasma melatonin secretion in the luteal phase could be significantly increased compared to the follicular phase in humans (Webley and Leidenberger, 1986), so melatonin may affect the change in clock genes between the follicular phase and luteal phase in STH sheep. Although the expression of clock genes in the luteal phase is higher than that in the follicular phase, there is no significant difference in some tissues, perhaps due to the relatively small sample size.

## 5 Conclusion

In conclusion, this study describes the expression pattern of six canonical clock genes in the follicle and luteal phases of STH sheep. All six genes were expressed in both reproductive and non-reproductive tissues of different phases, with high expression levels shown in the luteal phase. Our results suggest that the estrous cycle has an impact on clock gene expression. However, further studies are needed to elucidate the changes in clock gene expression in the estrous cycle and their biological role during this process. This is the first study performed on the tissue-specific expression patterns of the six canonical clock genes in the follicle and luteal phases of STH sheep, providing a foundation for elucidating the molecular mechanism underlying the effect of clock genes on the ewe estrous mode.

## Data Availability

Data are available upon request from the corresponding author.
